# Vancomycin continuous infusion versus intermittent infusion during continuous venovenous hemofiltration: slow and steady may win the race

**DOI:** 10.1186/s13613-015-0048-x

**Published:** 2015-05-08

**Authors:** Hsin Lin, Yana Bukovskaya, Marc De Moya, Jarone Lee, Ulrich Schmidt

**Affiliations:** Department of Pharmacy, Massachusetts General Hospital, 55 Fruit Street, Boston, MA 02114 USA; Department of Pharmacy, Veterans Affairs Greater Los Angeles Healthcare System, 11301 Wilshire Blvd., Los Angeles, CA USA; Division of Trauma, Emergency Surgery and Surgical Critical Care, Massachusetts General Hospital, 55 Fruit Street, Boston, MA 02114 USA; Departments of Surgery and Emergency Medicine, Massachusetts General Hospital, 55 Fruit Street, Boston, MA 02114 USA; Department of Anesthesiology, University of California, 200 West Arbor Drive, San Diego, CA 92103 USA

**Keywords:** Vancomycin, Continuous infusion, Continuous venovenous hemofiltration

## Abstract

**Background:**

Vancomycin during continuous venovenous hemofiltration (CVVH) is either administered by intermittent infusion (II) or continuous infusion (CI). In this patient population, the best method to rapidly achieve target serum concentrations of 15 mcg/ml to 25 mcg/ml remains to be elucidated. We hypothesized that CI would achieve a target serum level of 15 mcg/ml to 25 mcg/ml within 24 h of the initiation of therapy more consistently than II.

**Methods:**

A retrospective cohort study of adult patients admitted to the intensive care unit (ICU) between 2011 and 2014 receiving intravenous vancomycin with 24-hour serum level while on CVVH was included. Patients were excluded from this review if they had residual renal function during CVVH, were concomitantly on extracorporeal membrane oxygenation, or if the first dose of vancomycin was received six or more hours prior to the initiation of CVVH. The primary outcome was the achievement of a therapeutic level of 15mcg/ml to 25 mcg/ml by 24 hours.

**Results:**

Fifty-nine patients met the inclusion criteria and 14 received CI and 45 in II. Therapeutic 24-hour levels were achieved in 14/14 versus 2/45 in CI and II, respectively (*p* < 0.001). Mean 24-hour vancomycin levels were 20.35 ± 2.78 mcg/ml for CI compared to 9.7 ± 3.52 mcg/ml for II (*p* < 0.001). Mean loading dose was 26.65 ± 3.06 mg/kg for CI compared to 17.58 ± 5.72 mg/kg for II (*p* < 0.001). Daily maintenance doses were 15.66 ± 6.26 mg/kg for CI compared to 17.28 ± 4.96 mg/kg for II (*p* = 0.339). In the subgroup of 27 patients who received vancomycin-loading dose >20 mg/kg, mean 24-hour levels were 20.35 ± 2.78 mcg/ml for CI versus 11.8 ± 2.7 mcg/ml for II (*p* < 0.001). No significant differences were found between patients in the two groups with respect to CVVH rate and length of CVVH prior to vancomycin administration.

**Conclusions:**

The results of our study suggest that critically ill patients on CVVH treated with CI achieved the target level faster than II and consistently keep the vancomycin level within target range.

## Background

Continuous renal replacement therapy (CRRT) is a frequently used modality for critically ill patients with severe acute kidney injury who cannot tolerate intermittent hemodialysis due to hemodynamic instability [1]. Acute kidney injury occurs in up to 50% of septic patients, and 70% of these patients will require CRRT [2]. An ongoing concern for these patients is the high incidence of infection and associated mortality that have been reported to be as high as 60% [3]. Optimizing antibiotic dosing is a priority in these patients since inadequate systemic concentrations may lead to treatment failure and development of antimicrobial resistance, while excessive concentrations may lead to drug toxicity [4].

Subtherapeutic vancomycin levels are not uncommon while receiving CRRT [5]. Furthermore, the study of vancomycin therapy while on CRRT was based on steady-state serum levels, which may not represent the therapeutic effect required in the early phase of sepsis [5]. Reliance upon steady-state drug concentrations ignores events occurring while the pathogen is exposed to intermittent subtherapeutic drug concentrations prior to steady state. It is essential to ensure that the appropriate PK/PD target concentration is achieved promptly within the first 24 hours of antimicrobial therapy rather than delay this goal until steady state because the suboptimal exposure in the interim can potentially promote resistant bacterial strains [6]. In addition, several studies have demonstrated the important effects of early antimicrobial treatment on outcome [7]. In patients with acute severe renal failure, the best method to rapidly achieve target serum concentrations is unclear. The objective of our study was to evaluate whether traditional intermittent infusion (II) or continuous infusion (CI) of vancomycin results in appropriate serum concentrations in intensive care unit (ICU) patients within 24 hours.

## Methods

This retrospective cohort study examined adult ICU patients at an urban tertiary academic medical center who received concurrent continuous venovenous hemofiltration (CVVH) and vancomycin between January 2011 and August 2014. The institution review board of Massachusetts General Hospital approved the study and waived the need for informed consent. A computerized list of patients who received intravenous vancomycin and CVVH dialysates was generated by the Pharmacy Informatics Department through retrospective query of the electronic medication database, which allowed the identification of potential study patients. Vancomycin dosing was dictated by the treating clinician, and therapy could be administered either *via* II or CI. Patients were then grouped based on the mode of vancomycin infusion they received. First vancomycin dose is referred to as loading dose for the purpose of the analysis.

The study included critically ill adults aged >18 years undergoing CVVH and concomitantly receiving intravenous vancomycin with at least one level drawn 24 hours after the first dose. Patients were excluded if they had the following criteria: (1) an average urine output greater than 0.5 ml/kg/hr for six consecutive hours during the study, (2) were concomitantly on ECMO (3), or if the first dose of vancomycin was received six or more hours prior to the initiation of CVVH. All CVVH treatments applied the CAR-505 filter with a polyethersulfone membrane with a 1.6 m^2^ membrane surface area in conjunction with the NxStage System One dialysis machine. Goal vancomycin trough concentrations for II were in accordance with the Infectious Disease Society of America guideline recommendations, with 15 mcg/ml to 20 mcg/ml for severe infection [8]. Goal vancomycin-plateau concentration for CI was 15 mcg/ml to 25 mcg/ml based on published data [9]. Vancomycin concentration analysis utilized a Syva Emit 2000 vancomycin assay (Simens Healthecare Diagnostics, Inc. Newark, DE). The assay has an analytical range between 5.0 mcg/ml and 50.0 mcg/ml, and the between-run coefficient of variation was <10% throughout the analytical range.

Demographic data (age, sex, actual body weight, body mass index (BMI), baseline renal function, and comorbid conditions), severity of illness score (APACHEII) calculated at the time of ICU admission, Sequential Organ Failure Assessment score (SOFA score) at the commencement of vancomycin, and laboratory parameters were retrospectively collected for each patient in the study. Other gathered data included primary ICU service, CVVH rate, vancomycin dose, and serum vancomycin level. CI vancomycin clearance while on CVVH was calculated using the following equation:

$$ \frac{\mathrm{dose}\left(\mathrm{mg}/\mathrm{hr}\right)}{\mathrm{serum}\kern0.24em \mathrm{concentration}\left(\mathrm{mg}/\mathrm{L}\right)}=\mathrm{vancomycin}\kern0.24em \mathrm{clearance}\left(\mathrm{L}/\mathrm{hr}\right) $$

### Vancomycin administration and determination of serum-vancomycin concentration

Before 2013, the standard operating procedure for intravenous vancomycin in our ICU was the intermittent infusion of vancomycin with doses being at the discretion of the treating ICU physician to achieve the target serum vancomycin trough concentration of 15 mcg/ml to 20 mcg/ml. The usual starting dose was 1,000 mg to 2,000 mg of vancomycin daily. The serum-vancomycin levels were determined every day or at physician’s discretion. The maintenance dose was then adjusted to obtain a serum-vancomycin trough concentration of 15 mcg/ml to 20 mcg/ml and typically infused once daily [10].

Starting at the beginning of 2013, the standard procedure for intravenous vancomycin administration for patients who receive CVVH was changed to continuous infusion of vancomycin. After a loading dose of 25 mg/kg, vancomycin 1,500 mg (60 mg/hr) daily was continuously infused. The infusion rate was then adjusted to obtain a serum-vancomycin concentration of 15 mcg/ml to 25 mcg/ml every 24 hours. When there were two consecutive serum concentrations in the target range and no change in CVVH flow rate, vancomcycin concentration is no longer monitored.

#### Endpoints

The primary outcome was the achievement of a therapeutic level by 24 hours. Secondary outcomes were time to therapeutic level and 24-hour level. Therapeutic levels were defined as a range of 15 mcg/ml to 25 mcg/ml for both II and CI. An *a priori* determined subgroup analysis of patients who received a greater than 20 mg/kg first dose was performed, and primary, secondary endpoints and other clinical outcomes were analyzed comparing the CI and II group.

Safety endpoint: hemodialysis upon discharge from the hospital, supratherapeutic vancomycin levels (>25mcg/ml).

#### Data analysis

Distributions of quantitative outcomes were summarized by mean with standard deviation and compared by using the Mann-Whitney U test, and those of categorical outcomes were summarized by counts and proportions and compared by using the chi-square test (or Fisher’s exact test as appropriate). A sample of 40 patients was required to achieve a power of 80% to detect a 50% absolute difference in the primary outcome at a significant level of 5%, with a target of 13 in the CI group and 27 in the II group. All analyses were carried out using STATA Data Analysis and Statistical Software (version 13, StatCorp LP, College Station, TX). A *p* value of <0.05 was considered statistically significant.

## Results

**Figure 1:**
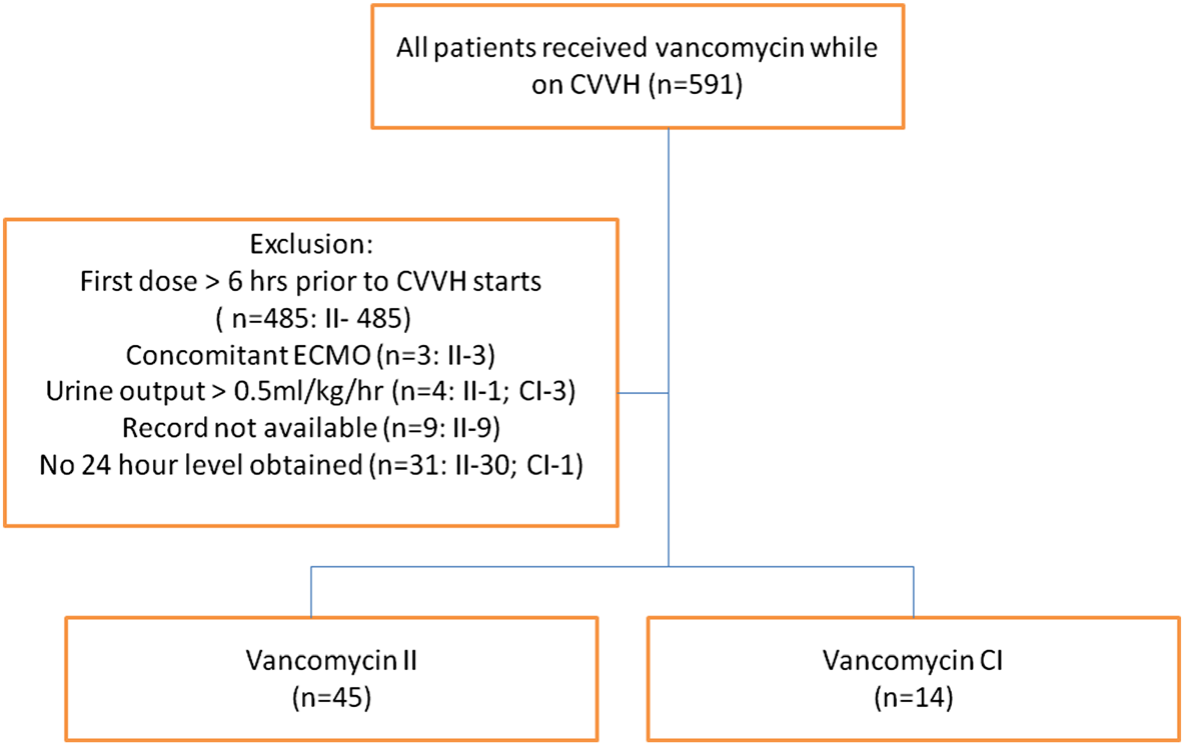
Study flow diagram.

During the study period, 591 patents underwent evaluation for eligibility (Figure 1). Fifty-nine patients met inclusion criteria, 14 received CI and 45 received II. The 59 included patients had a mean age of 64 ± 14.8 years, and 71.2 % were male. Baseline characteristics were significantly different for admitting APACHEII score, and more patients in the CI group were admitted as surgical ICU patients and had a significant longer length of stay prior to CVVH initiation in the CI group (Table 1). There was no difference in SOFA score at the initiation of vancomycin therapy. Hemofiltration rates were 23.75 ± 8.08 ml/kg/hr and 22.1 ± 7.7 ml/kg/hr in the CI and II groups, respectively (*p* = 0.501). CVVH duration was 262 ± 97.58 hr and 243.2 ± 227.4 hr in the CI and II groups, respectively (*p* = 0.761).

**Table 1:** **Baseline patient characteristics and demographic data**

	**Vancomycin CI (n= 14)**	**Vancomycin II (n= 45)**	***p***
Age, year	60.64 (17.91)	64.3 (13.95)	0.417
Gender, male no. (%)	9 (64.2)	33 (73)	0.513
BMI, kg/m2	31.08 (6.63)	31.1(7.64)	0.969
BMI > 30 kg/m2, *n* (%)	6 (42.8)	21 (46.6)	0.803
Weight (kg)	92.14 (23.39)	91.0 (27.1)	0.890
CKD baseline, *n* (%)	6 (42.8)	21 (46.6)	0.803
HD baseline, *n* (%)	3 (21.4)	14 (31.1)	0.485
Mechanical ventilation*, *n* (%)	14 (100)	43 (95.5)	0.422
Vasopressor therapy*, *n* (%)	13 (92.8)	40 (88.8)	0.668
APACHEII	26.42 (8.01)	32.13(7.69)	0.019
SOFA score at the onset of therapy	14.5 (2.028)	16.5 (0.327)	0.880
ICU patients, *n* (%)			0.002
SICU	13 (91.6)	21 (46.6)	
MICU	1 (8.4)	24 (53.4)	
Albumin (g/dl)	3.01 (0.72)	2.86 (0.58)	0.431
LOS prior to CVVH (hr)	90.85 (114.97)	38.58 (62.55)	0.03
Hospital mortality	7 (50)	26 (57.7)	0.609
CVVH hemofiltration rate (ml/kg/hr)	23.75 (8.08)	22.1 (7.7)	0.501
CVVH hemofiltration rate >30 ml/kg/hr, *n* (%)	4 (28.5)	7 (15.5)	0.275
CVVH duration (hr)	262 (97.58)	243.1 (227.4)	0.761
Vancomycin start in relation to CVVH (day)	1.39 (1.84)	2.95 (5.62)	0.313

LOS, length of stay. Value expressed as means (standard deviation), unless otherwise specified.

*Need for mechanical ventilation, vasopressor therapy when vancomycin was started.

### Endpoints

**Figure 2:**
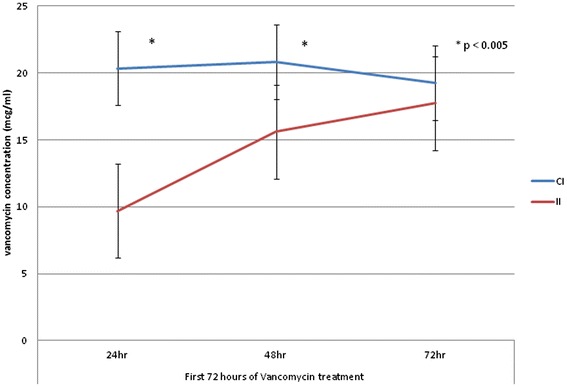
Vancomycin concentrations on the first 72 hours of therapy between CI and II.

Regarding the primary endpoint, a therapeutic 24-hour level was achieved in 14/14 versus 2/45 in CI and II, respectively (*p* < 0.001) (Table 2). Mean 24-hour vancomycin levels were 20.35 ± 2.78 mcg/ml for CI compared to 9.7 ± 3.52 mcg/ml for II (*p* < 0.001). The mean loading dose was 26.65 ± 3.06 mg/kg for CI compared to 17.58 ± 5.72 mg/kg for II (*p* < 0.001). Total dose prior to first 24-hour levels were 3625 ± 497.5 mg for CI compared to 2,000 ± 310 mg for II (*p* < 0.001). Of note, total dose prior to first 24-hour level for CI includes both loading dose and first 24-hour maintenance infusion, which immediately initiated post-loading dose. Daily maintenance doses were 15.66 ± 6.26 mg/kg for CI compared to 17.28 ± 4.96 mg/kg for II (*p* = 0.339). The time to achieve therapeutic levels was 24.0 hours and 59.6 ± 21.6 hours in the CI and II groups (*p* < 0.001). Significantly higher daily vancomycin levels were observed in the CI group than in the II group for the first and second days of therapy (Figure 2). No difference in hospital mortality was noted between therapeutic level achievers and nonachievers (*p* = 0.629).

**Table 2:** **Vancomycin treatment and vancomycin serum level**

	**Vancomycin CI (n= 14)**	**Vancomycin II (n= 45)**	***p***
Duration of therapy (day)	6.57 (3.25)	7.31 (3.34)	0.470
Loading dose (mg)	2232.14 (385.61)	1533.33 (565.58)	<0.001
Loading dose (mg/kg)	26.65 (3.06)	17.58 (5.72)	<0.001
Daily maintenance dose (mg/kg)	15.66 (6.26)	17.28 (4.96)	0.339
Maintenance dose - day 1 (mg)	1329.85 (335.61)	1328.6 (1286.47)	0.381
Maintenance dose - day 2 (mg)	1339.28 (348.17)	1585.68 (538.83)	0.116
Maintenance dose - day 3 (mg)	1428.57 (301.09)	1558.17 (495.61)	0.374
Total dose prior to first 24-hour level (mg)	3625 (497.5)	2000 (310.1)	<0.001
24-hour level (mcg/ml)	20.35 (2.78)	9.7 (3.52)	<0.001
48-hour level (mcg/ml)	20.8 (2.66)	15.61 (4.64)	0.001
72-hour level (mcg/ml)	19.25 (2.01)	17.74 (3.02)	0.201
24-hour level 15 mcg/ml to 25 mcg/ml, *n* (%)	14 (100)	2 (4.4)	<0.001
Time to therapeutic level (hr)	24 (0.56)	59.6 (21.6)	<0.001
Determination of serum concentration per day of vancomycin therapy, *n*	0.45 (0.24)	0.56 (0.21)	0.264
Determination of serum concentration, total per patient, *n*	2.62 (0.74)	3.26 (1.03)	0.136

For CI group, maintenance dose starts immediately after loading.

Value expressed as means (standard deviation).

In the subgroup of 27 patients (14 in CI and 13 in II) who received a vancomycin loading dose >20 mg/kg, mean 24-hour levels were 20.35 ± 2.78 mcg/ml for CI versus 11.8 ± 2.7 mcg/ml for II (*p* < 0.001). the time to therapeutic level was 24 hours for CI versus 48.9 ± 17.6 hr for II (*p* < 0.001) (Table 3). Therapeutic 24-hour level was achieved in 14/14 versus 1/13 in CI and II, respectively (*p* < 0.001). The mean loading dose was 26.65 ± 3.06 mg/kg for CI compared to 24.4 ± 2.86 mg/kg for II (*p* = 0.082). The total dose prior to first 24-hour levels was 3,625 ± 497.5 mg for CI compared to 1,846.15 ± 665.66 mg for II (*p* < 0.001).

**Table 3:** **Vancomycin treatment and serum level for loading dose > 20 mg/kg**

	**Vancomycin CI (n= 14)**	**Vancomycin II (n= 13)**	***p***
Loading dose (mg)	2232.14 (385.61)	1846.15 (665.66)	0.074
Loading dose (mg/kg)	26.65 (3.06)	24.44 (2.86)	0.082
Total dose prior to first 24-hour level (mg)	3625 (497.5)	1846.15 (665.66)	<0.001
Total dose prior to first 24-hour level (mg/kg)	43.01 (8.31)	24.1 (2.57)	<0.001
Maintenance dose - day 1 (mg)	1329.85 (335.61)	1623.07 (177.05)	0.231
Maintenance dose - day 2 (mg)	1339.28 (348.17)	1455 (454.54)	0.456
Maintenance dose - day 3 (mg)	1428.57 (301.09)	1496.6 (377.98)	0.628
24-hour level (mcg/ml)	20.35 (2.78)	11.8 (2.7)	<0.001
24-hour level 15 mcg/ml to 25 mcg/ml, *n* (%)	14 (100)	1 (7%)	<0.001
Time to therapeutic level (hr)	24 (0.56)	48.9 (17.6)	< 0.001

For CI group, maintenance dose starts immediately after loading.

Value expressed as means (standard deviation).

**Figure 3:**
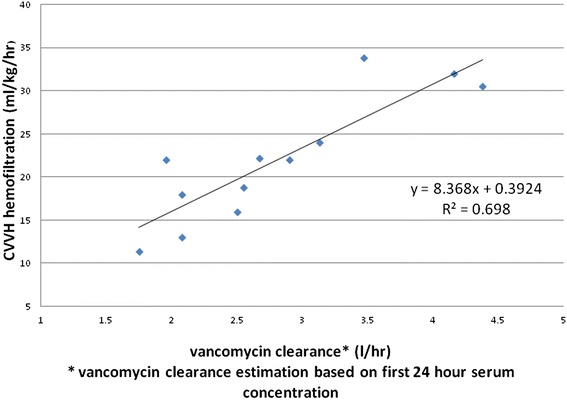
Correlation of CI vancomucin clearance and CVVH hemofiltration.

A significant positive correlation between hemofiltration rate and vancomycin clearance was observed in the CI group (Figure 3). Mean vancomycin clearance was 2.88 ± 0.81 l/hr. The regression equation generated for vancomycin clearance and CVVH rate is as follows:

$$ \frac{\mathrm{hemofiltration}\kern0.24em \mathrm{rate}\frac{\frac{\mathrm{ml}}{\mathrm{kg}}}{\mathrm{hr}}\hbox{-} 0.392}{8.368}=\mathrm{vancomycin}\kern0.24em \mathrm{clearance}\left(\mathrm{L}/\mathrm{hr}\right) $$

**Figure 4:**
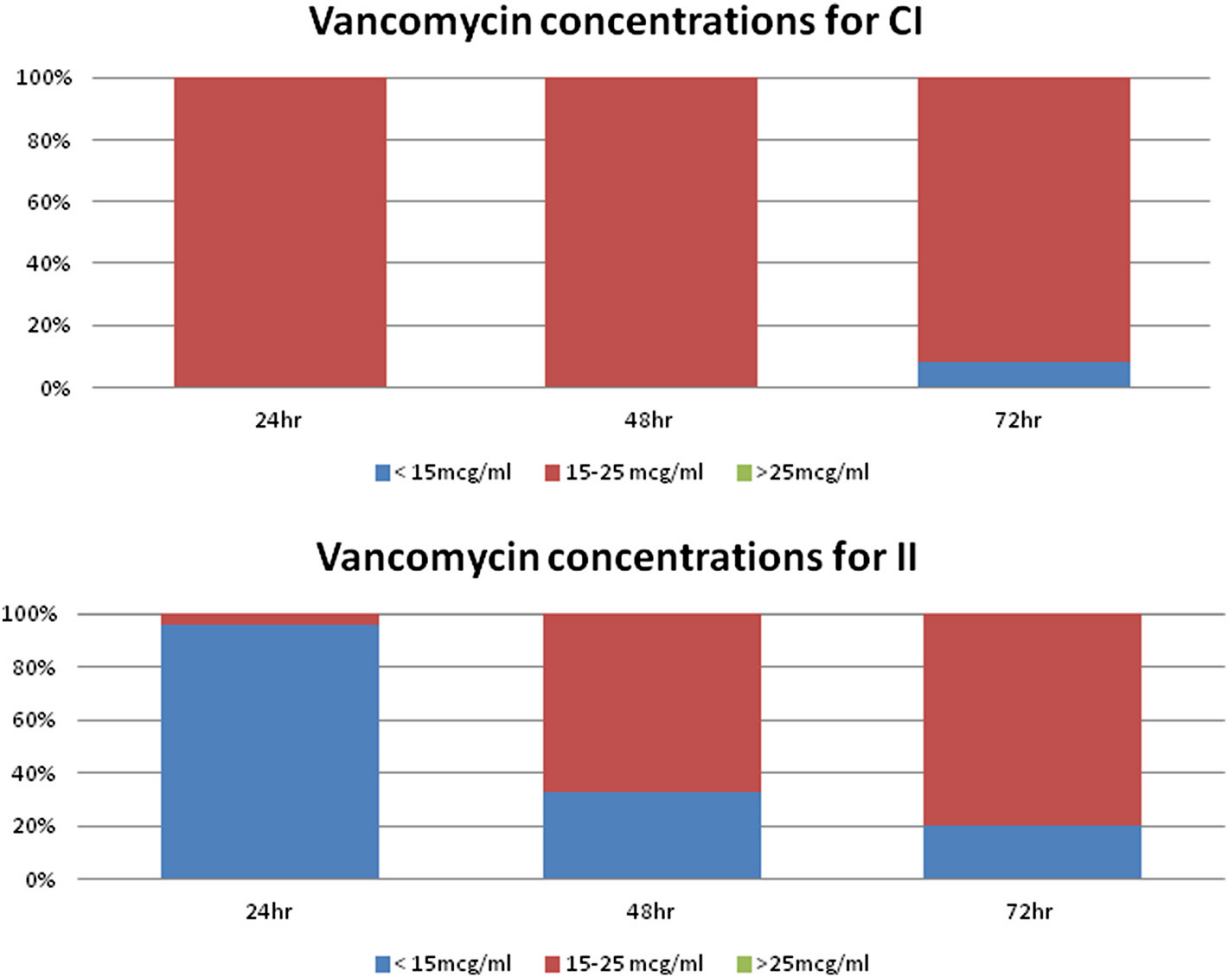
Proportion of patients with inadequate (<15mcg/ml), adequate (15-25mcg/ml), and excessive (>25mcg/ml) concentrations on the first 72 hours of therapy.

There was no evidence of a correlation between patient weight and the daily maintenance doses needed to achieve a therapeutic level. No patient in either group had a supratherapeutic vancomycin level (>25 mcg/ml) during treatment (Figure 4). One patient in the II developed acute kidney and required hemodialysis upon discharge from the hospital. None of the patients developed hearing loss.

## Discussion

To our knowledge, this is the first study comparing the 24-hour target attainment between CI versus II utilizing the same CVVH system. The present study revealed that a substantial proportion of patients in the II group did not achieve the target vancomycin level, while all the patients in the CI group achieved target vancomycin level within 24 hours of therapy. In order to control for the loading dose difference, an *a priori* subgroup analysis was done which demonstrated that in all patients who received at least 20 mg/kg loading dose, CI still achieved a therapeutic serum level significantly more often than II within 24 hours. Previous prospective studies have suggested that vancomycin when administered as CI is a more effective method in achieving early serum concentration targets [11]. Unlike the study by Wysocki, our study had a very different ICU population than that of those patients who have impaired renal function and required CVVH. Our finding demonstrates that an average of at least an additional 24 hours was needed for the II group to achieve target concentration compared to the CI group.

In the subgroup patients who received a >20 mg/kg loading dose, total doses received prior to 24-hour levels were 3,725 mg (43.01 mg/kg) in the CI and 1,846 mg (24.1 mg/kg) in the II. We cannot rule out the possibility that in the II when given the same vancomycin dose, a similar goal would have been achieved at 24 hour as CI. However, the safety profile of as high as 40 mg/kg single dose, the comparable dose used in the CI has not been established.

Consistent with a previous study, we observed a higher variability in the antibiotic serum levels in critically ill patients receiving vancomycin II than CI while on CVVH [5]. This might be explained by significant changes in vancomycin pharmacokinetics during critical illness, such as increased volume of distribution and varying vancomycin clearance during CVVH. A continuous infusion steady-state concentration (mg/l) that follows linear pharmacokinetics and vancomycin clearance can be calculated as:

$$ \frac{\mathrm{Maintenance}\kern0.24em \mathrm{infusion}\kern0.24em \mathrm{rate}\left(\mathrm{mg}/\mathrm{hr}\right)}{\mathrm{Vancomycin}\kern0.24em \mathrm{clearance}\kern0.24em \mathrm{b}\mathrm{y}\kern0.24em \mathrm{CVVH}\left(\mathrm{L}/\mathrm{hr}\right)}={\mathrm{CI}}_{\mathrm{ss}}\left(\mathrm{mg}/\mathrm{L}\right) $$

Despite the results of several studies that have concluded that an area under the curve (AUC)/minimum inhibitory concentration (MIC) ratio above 400 is required to achieve successful treatment for Methicillin-resistant Staphylococcus aureus (MRSA) infection [12-14], the general recommendations for MRSA infections are vancomcycin II with a target trough of 15 mcg/ml to 20 mcg/ml. It has recently been proposed to check two levels of vancomycin and calculate the AUC because a single time-point trough does not represent the concentration time curve that is associated with efficacy [15]. On the other hand, continuous infusion seems to be a justifiable route of administration because it takes into account the time-dependent pharmacodynamic effect of vancomycin. Furthermore, AUC for patients in CI can be easily estimated as follows:

$$ {\mathrm{AUC}}_{24\mathrm{hr}}=\mathrm{serum}\kern0.24em \mathrm{plateau}\kern0.24em \mathrm{c}\mathrm{oncentration}\left(\mathrm{m}\mathrm{c}\mathrm{g}/\mathrm{ml}\right)\times 24\mathrm{hr} $$

Therefore, by keeping the serum-plateau concentration between 15 mcg/ml to 25 mcg/ml, AUC_24_ can be consistently achieved at above 400.

Previous work by Beumier et al. showed higher loading doses followed by continuous infusion allowed for the rapid achievement of target vancomycin concentrations in the majority of patients [9]. The CI vancomycin regimen used in the study includes a loading dose of 35 mg/kg followed by a CI of 14 mg/kg over 24 hours. Another recent study showed that a wide range of daily doses, 16mg/kg to 35 mg/kg, was necessary to provide adequate vancomycin concentrations in most study patients [16]. Our data supports that the CI maintenance-dosing regimen should be based on CVVH flow rate because a positive correlation was found between the intensity of CVVH and vancomycin clearance in patients who received CI. In other words, the more intense the hemofiltration rate, the higher the vancomycin continuous infusion dose is needed to achieve target serum concentration. Previous work by Frazee et al. showed an inverse correlation between hemofiltration rate and serum vancomycin concentration (*r* = − 0.423, *p* < 0.001) [17]. We used the following equation to calculate daily vancomycin dose needed to obtain a target concentration of 20 mcg/ml while on continuous infusion:

$$ \mathrm{target}\kern0.24em \mathrm{concentration}\kern0.24em 20\left(\frac{\mathrm{mg}}{\mathrm{L}}\right)\times \mathrm{drug}\kern0.24em \mathrm{clearance}\kern0.24em \mathrm{rate}\left(\frac{\mathrm{L}}{\mathrm{hr}}\right)\times 24\mathrm{hours} $$

This allows the clinician to calculate varying maintenance-dose regimens based on ultrafiltration rate and duration. Furthermore, this assists dose adjustment easily, as clinically CRRT parameters may be modified many times in the course of antibiotic therapy. Vancomycin clearance for II was not assessed in our study because our standard practice was to obtain trough for vancomycin, and therefore, clearance estimation was not available.

Vancomycin-associated toxicities are mainly nephrotoxicity and ototoxicity. None of the patients developed hearing loss. Our study did not find any difference in patients discharged with new requirement of hemodialysis between groups. Conflicting results have been shown in renal toxicity associated with continuous infusion. Spapen et al. suggested a substantial increase in acute kidney injury at vancomycin plateau levels >25 mcg/ml during continuous infusion, and likely, the high serum levels are responsible for the renal toxicity [18]. On the other hand, Hanrahan et al. showed that a continuous infusion method is associated with significantly less nephrotoxicity than dosing by intermittent infusion [19]. Our study is not powered to detect differences in renal function recovery, however, supratherapeutic vancomycin concentration was not observed in either group.

Our study has several limitations mainly due to its retrospective nature. First, the adequacy of loading was not assessed. It is possible that critically ill patients undergoing CVVH would merit a larger than recommended loading dose. However, no post-loading serum levels were available for analysis. Second, a potential selection bias exists due to the large portion of patients excluded due to the absence of an available concentration or having received vancomycin more than six hours prior to CVVH start. Third, time elapsed from hospital admission to vancomycin administration could be another confounder. Although the vancomycin II group had significant higher APACHEII scores at admission compared to the CI group, there were no differences in SOFA scores at the initiation of vancomycin therapy. It was not possible to rule out the potential pathophysiological disturbances that may affect antibiotic pharmacokinetics [20]. Fourth, our study result was based on a small sample size and has a higher chance of type-II error, however, we were powered for our findings. Fifth, we did not record data on microbiological response to vancomycin. Thus, to determine if whether optimizing antibiotic concentrations during CVVH leads to improved outcomes requires further study. Sixth, our study extends over three years, and we were not able to take into account for the natural influence in the practice pattern. Lastly, we evaluated only one CRRT condition. Multiple variables associated with various extracoporeal systems would affect drug removal; therefore, our dosing recommendation can only apply to CVVH and limit the generalization of these results to other CRRT modes, such as continuous venovenous hemodialysis (CVVHD) and continuous venovenous hemodiafiltration (CVVHDF).

## Conclusions

The results of our study suggest that critically ill patients on CVVH treated with CI achieved the target level faster than II in 24 hours and consistently keep the vancomycin level within target range.

## Abbreviations

CVVH: continuous venovenous hemofiltration
CI: continuous infusion
II: intermittent infusion
CRRT: continuous renal replacement therapy
BMI: body mass index
APACHEII: Acute physiological and Chronic Health Score
AUC: area under the curve
MIC: minimum inhibitory concentration
SOFA: sequential organ failure assessment
CVVHD: continuous venovenous hemodialysis
CVVHDF: continuous venovenous hemodiafiltration
